# α-Glucosidase and tyrosinase inhibitory effects of an abietane type diterpenoid taxoquinone from *Metasequoia glyptostroboides*

**DOI:** 10.1186/s12906-015-0626-3

**Published:** 2015-03-26

**Authors:** Vivek K Bajpai, Yong-Ha Park, MinKyun Na, Sun Chul Kang

**Affiliations:** Department of Applied Microbiology and Biotechnology, School of Biotechnology, Yeungnam University, Gyeongsan, Gyeongbuk 712-749 Korea; Department of Biotechnology, Daegu University, Gyeongsan, Gyeongbuk 712-714 Korea; College of Pharmacy, Chungnam National University, Daejeon, 305-764 Korea

**Keywords:** *Metasequoia glyptostroboides*, Taxoquinone, α-Glucosidase, Tyrosinase, Terpenoids

## Abstract

**Background:**

Nowadays plant derived natural compounds have gained huge amount of research attention especially in food and medicine industries due to their multitude of biological and therapeutic properties as alternative medicines.

**Methods:**

In this study, a diterpenoid compound taxoquinone, isolated from *Metasequoia glyptostroboides* was evaluated for its α–glucosidase and tyrosinase inhibitory efficacy in terms of its potent anti-diabetic and depigmentation potential, respectively.

**Results:**

As a result, taxoquinone at the concentration range of 100–3,000 μg/mL and 200–1,000 μg/mL showed potent efficacy on inhibiting α-glucosidase and tyrosinase enzymes by 9.24-51.32% and 11.14-52.32%, respectively.

**Conclusions:**

The findings of this study clearly evident potent therapeutic efficacy of an abietane diterpenoid taxoquinone isolated from *M. glyptostroboides* with a possibility for using it as a novel candidate in food and medicine industry as a natural alternative medicine to prevent diabetes mellitus type-2 related disorders and as a depigmentation agent.

## Background

Diabetes mellitus is a metabolic disorder caused by a lack of insulin characterized by hyperglycemia [1]. Diabetes mellitus type 2 is also known as non-insulin-dependent diabetes mellitus which is caused by insulin dysfunction, especially after food intake. The effective treatment for type 2 is to inhibit or delay intestinal carbohydrate digestion. Carbohydrates which are the major components of our daily foods, for instance polysaccharides, are transformed into simple sugars, and then absorbed through the intestine. α-Glucosidase, an enzyme located in the small intestine epithelium, catalyzes the cleavage of disaccharides and oligosaccharides to glucose. Glucosidase inhibitors reduce the rate of carbohydrate digestion and delay the carbohydrate absorption from the digestive tract. Therefore, they have a potential to prevent the development of type 2 diabetes mellitus by lowering the after-meal glucose levels [2].

Human skin contains four major chromophores including haemoglobin, oxyhaemoglobin, carotenoids and melanin(s) where melanin acts as a dominant component of normal skin color and pigmentation [3]. The epidermis cells in the innermost layer of skin produce melanin upon exposure of ultraviolet radiation leading to tyrosinase mediated melanogenesis [3]. Tyrosinase is a multifunctional copper-containing enzyme, widely distributed in plants and animals which catalyzes the initial step in the formation of the pigment melanin from tyrosine [4]. Hence, tyrosinase is known to be a key enzyme for melanin biosynthesis in plants and animals. Although use of tyrosinase inhibitors such as kojic acid and hydroquinone is gaining increasing attention in the cosmetic industry due to their anti-pigmenting effects, they have found to exert severe causes of skin inflammations. Hence, use of natural plant based secondary metabolite to cure skin diseases especially for cosmetic purposes could be a safe and alternative therapy in cosmetic industry to provide lead compounds to serve as natural anti-pigmentation compounds [5].

Phytochemicals confer various health benefits, among them, α-glucosidase and tyrosinase inhibitory activities have particularly received intensive attention due to the increasing number of patients suffering from diabetes type 2 and skin disorders. Though synthetic α-glucosidase and tyrosinase inhibitors have been used effectively, many doubts have been raised on their safety such as increased toxicity and adverse side effects. Hence, attention has been focused on the effective use of plant based compounds which are less toxic and natural in origin.

Although biological and therapeutic potential of *M. glyptostroboides* derived compounds has been reported previously [6-8], no report is available on α-glucosidase and tyrosinase inhibitory effects of taxoquinone, a diterpenoid from *M. glyptostroboides*. Hence, the aim of this research is to confirm the therapeutic potential of taxoquinone as a potent α-glucosidase and tyrosinase inhibitor.

## Methods

### Chemicals and instrument

Kojic acid, acarbose, sodium azide (NaN_3_) bovine serum albumin, P-Nitrophenyl-α-D-glucopyranoside, yeast α-glucosidase, mushroom tyrosinase, and 3,4-dihydroxy-L-phenylalanine (DOPA) were purchased from (Sigma-Aldrich, St. Louis, MO, USA). All other reagents used were of high analytical grade. Spectrophotometric measurements were done by using a 96-well micro-plate ELISA reader (Infinite M200, Tecan, Switzerland).

### Plant material

The mature cones (16 ~ 22 months old) of *M. glyptostroboides* were collected from Pohang city, Korea, and identified by the morphological features and the database present in the library at the Department of Biotechnology, Daegu University, Korea. A voucher specimen of *M. glyptostroboides* cone (DUB-0038) was deposited in the herbarium of College of Engineering, Department of Biotechnology, Daegu University, Korea.

### Extraction and isolation of taxoquinone

Dried cones of *M. glyptostroboides* (2 kg) were milled into powder and then extracted with ethyl acetate at room temperature for 12 days. The extract was evaporated under reduced pressure using a rotary evaporator (EYELA N1000, Japan). The dried ethyl acetate extract (7 g) was subjected to column chromatography over silica gel (mesh 230–400 mesh, Merck, Darmstadt, Germany) and was eluted with hexane-ethyl acetate-methanol solvent system to give 20 fractions. Of the fractions obtained, fraction-12 was further purified by preparative TLC over silica gel GF254 using hexane-ethyl acetate (1:2) as a mobile phase to give one compound (152 mg) which on the basis of spectral data analysis was characterized as a taxoquinone [6].

### Assay of α-glucosidase inhibition

α-Glucosidase inhibitory activity of taxoquinone isolated from *M. glyptostroboides* was evaluated according to the chromogenic method [9]. Briefly 10 μL of test samples at various concentrations (100, 500, 1,000, 2,000 and 3,000 μg/mL) and 50 μL of yeast α-glucosidase, dissolved in 100 mM phosphate buffer (pH 7.0) (containing 2 g/L bovine serum albumin and 0.2 g/L NaN_3_) were mixed in 96 well micro-plate and absorbance at 405 nm was measured for titer at zero time with a micro-plate reader (Tecan, Infinite M200, Mannedorf, Switzerland). After 5 min incubation, 50 μL of P-Nitrophenyl-α-D-glucopyranoside (5 mM) in the same buffer (pH 7.0) was used as a substrate solution and incubated for an additional 5 min at room temperature. Eventually the reaction was terminated by adding 80 μL of 0.2 M sodium carbonate solution. Absorbance of the reaction mixture was measured with a micro-plate reader at 405 nm. The increase in absorbance from zero time was measured. Inhibitory activity was expressed as 100 minus relative absorbance difference (%) of test compounds to absorbance change of the control, while the reaction system without sugiol was served as a control test. The system without α-glucosidase was used as blank, and acarbose at various concentrations (100, 500, 1,000, 5,000 and 10,000 μg/mL) was used as a positive control. Each experiment was conducted in triplicate, and the enzyme inhibitory rate was calculated as follows:

$$ \mathrm{Inhibition}\ \left(\%\right) = \left(\mathrm{Control}\ \mathrm{absorption} - \mathrm{Sample}\ \mathrm{absorption}\right)/\ \mathrm{Control}\ \mathrm{absorption} \times 100 $$

### Assay of tyrosinase inhibition

The tyrosinase activity of taxoquinone was measured by a previously reported method [10]. Briefly, 100 μL of different concentrations (200, 400, 600, 800 and 1,000 μg/mL) of taxoquinone were mixed with 600 μL of 0.175 M sodium phosphate buffer (pH 6.8). Further, 200 μL of 10 mM L-DOPA solution (L-3,4-dihydroxyphenyl-alanine) was added to each well. After that, 200 μL of tyrosinase (110 units/mL in 0.175 M sodium phosphate buffer) was added to the reaction mixture and further incubated at 37°C for 2 min. Then after incubation, the amount of dopachrome produced in the reaction mixture was measured at 475 nm in a 96-well micro-titer plate with a micro-plate reader. Kojic acid (20, 40, 100, 200 and 500 μg/mL) was used as a positive control. The experiment was conducted in triplicate at room temperature, and the enzyme inhibitory rate was calculated as follows:

$$ \mathrm{Inhibition}\ \left(\%\right) = \left(\mathrm{Control}\ \mathrm{absorption} - \mathrm{Sample}\ \mathrm{absorption}\right)/\ \mathrm{Control}\ \mathrm{absorption} \times 100 $$

### Statistical analysis

All the data were expressed as mean ± standard deviation of three replicates. Tests of significant differences were determined by one way ANOVA followed by Duncan’s test using SAS software (SAS 9.2, SAS), and the values were considered to be significant (p < 0.05).

## Results and discussion

### Identification of taxoquinone

**Figure 1:**
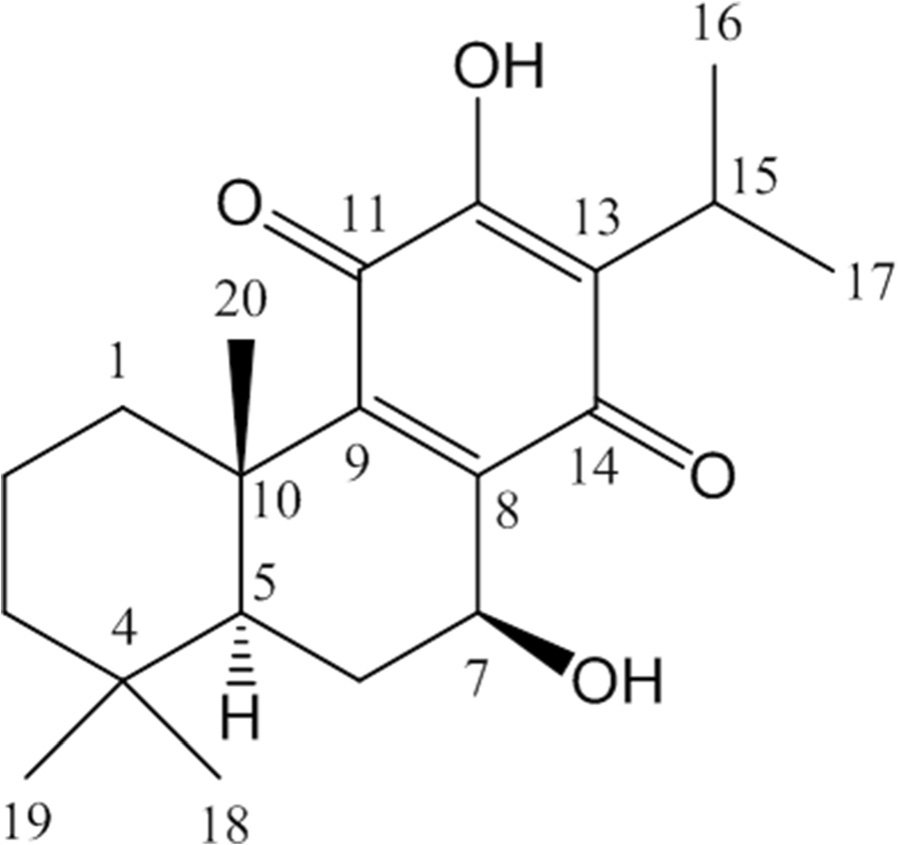
**Chemical structure of a diterpenoid taxoquinone isolated from** ***Metasequoia glyptostroboides*** **.**

The ethyl acetate cone extract of *M. glyptostroboides* after column chromatography over silica gel yielded a pure compound which was obtained as orange needles with a specific melting point (mp 212–214°C). The ^1^H NMR spectrum (CDCl_3_, 250 MHz) of the compound showed an oxygenated methine signal at δ_H_ 4.77 (1H, ddd, *J* = 2.2, 7.4, 9.8 Hz, H-7), a hydroxyl group at δ_H_ 3.80 (1H, d, *J* = 2.2 Hz, 7-OH), a tertiary methine signal at δ_H_ 3.14 (1H, sept, *J* = 7.1 Hz, H-15), three tertiary methyl signals at δ_H_ 1.33 (3H, s, H-20), 0.92 (3H, s, H-18), 0.90 (3H, s, H-19), and two secondary methyl signals at δ_H_ 1.20 (1H, d, *J* = 7.1 Hz, H-17), 1.18 (1H, d, *J* = 7.1 Hz, H-16). Further analysis of the ^1^H-^1^H COSY data established the connectivity through H-7 (δ_H_ 4.77), 7-OH (δ_H_ 3.80), and H-6b [δ_H_ 2.18 (1H, dd, *J* = 7.4, 12.5 Hz)], and through H-1b [δ_H_ 2.65 (1H, br d, *J* = 13.0 Hz)], H-2b [δ_H_ 1.71 (1H, br qt, *J* = 13.0, 3.0)], and H-1a, 2a, 3a, 3b (δ_H_ 1.65 – 1.01, m). In addition, two methyl signals at δ_H_ 1.20 (H-17) and 1.18 (H-16) correlated with a methine signal at δ_H_ 3.14 (H-15), which implicated an abietane-type diterpenoid. Twenty carbon signals including two carbonyl groups at δ_C_ 189.6 (C-14) and 183.7 (C-11) in the ^13^C NMR spectroscopic data strongly supported that this compound should be an abietane diterpenoid. On the basis of the interpretation of HMQC and HMBC data, this compound was proposed to be taxoquinone. By comparison of the multiplicity of H-7 and the chemical shifts both in the ^1^H and ^13^C NMR data, the structure of this compound (Figure 1) was determined to be taxoquinone [6].

### Inhibition of α-glucosidase

Phytochemicals efficiently contribute human being with health beneficial therapeutic agents of natural origin for their use in developing novel diabetes therapies or bio-therapeutic agents [11]. Although there is a huge ongoing interest in the application of plant-based compounds for alleviating chronic diseases due to their potent therapeutic and biomedicinal efficacy, the use of phytochemicals within the context of diabetes remains largely unexplored. This has urged the scientists to provide scientific evidences on the development of more effective agents conferring inhibitory effects on intestinal glucosidases. In this regard, inhibition of intestinal α-glucosidase to control hyperglycemia is an established strategy [11,12]. Hence, to overcome the adversary effects of clinical available synthetic α-glucosidase inhibitors, there is still need to develop alternative therapies to inhibit this key enzyme in order to minimize side effects and drug cost efficacy. A number of plant-based natural compounds have been screened for their enzymatic inhibitory activities [9,11,12].

**Figure 2:**
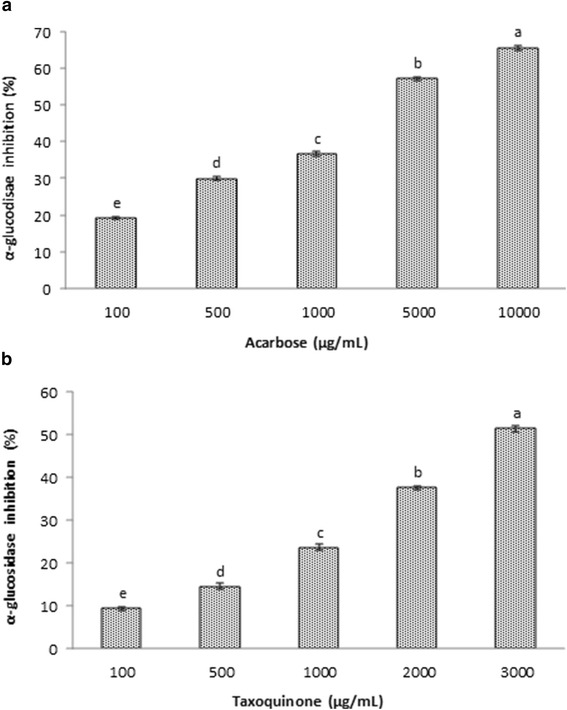
**α-Glucosidase inhibitory effect of standard compound acarbose (a) and taxoquinone (b) isolated from** ***Metasequoia glyptostroboides***. Data are expressed as mean ± SD (n = 3). Values with different superscripts are significantly different (p < 0.05).

In this assay, the α-glucosidase inhibitory activity of taxoquinone was found to be in a concentration dependent manner. The inhibitory effect of taxoquinone on α-glucosidase has been demonstrated in Figure 2. The taxoquinone at 100, 500, 1,000, 2,000 and 3,000 μg/mL showed the inhibition of α-glucosidase by 9.24, 14.43, 23.54, 37.43 and 51.32%, respectively. However, standard drug acarbose at 100, 500, 1,000, 5,000 and 10,000 μg/mL displayed α-glucosidase inhibitory effect by 19.16, 29.89, 36.68, 57.11 and 65.52%, respectively. In this assay, both the test compound taxoquinone and acarbose showed inhibitory effect in a dose-dependent manner. Similar findings on α-glucosidase inhibitory activity of flavonoid and terpenoid compounds isolated from *Agrimonia pilosa* were observed by Liu et al. [9]. In addition, sarcoviolins isolated from edible mushroom *Sarcodon leucopu*s were also found to inhibit α–glucosidase *in vitro* [12]. Also the terpenoids including hyptadienic acid isolated from the roots of *Potentilla fulgens* showed potent inhibitory effect on α–glucosidase *in vitro* [13]. Recently, three new phenylpropanoyl amides isolated from the leaves of *Piper sarmentosum* displayed remarkable α–glucosidase inhibitory activities [14].

### Inhibition of tyrosinase

Melanin is a polymerized natural coloring determinant widely distributed in plants, animals and microorganisms. A number of melanins are produced by multi-stepped enzymatic and non-enzymatic oxidation and polymerization processes. The mechanism of tyrosinase inhibition activity may be an important factor in the skin whitening of a cosmetic composition [15]. Melanin biosynthesis steps in the body include L-DOPA by tyrosine as a substrate, followed by its conversion to L-dopaquinone by the successive enzymatic oxidations, leading to take place a reaction of polymerization [15].

**Figure 3:**
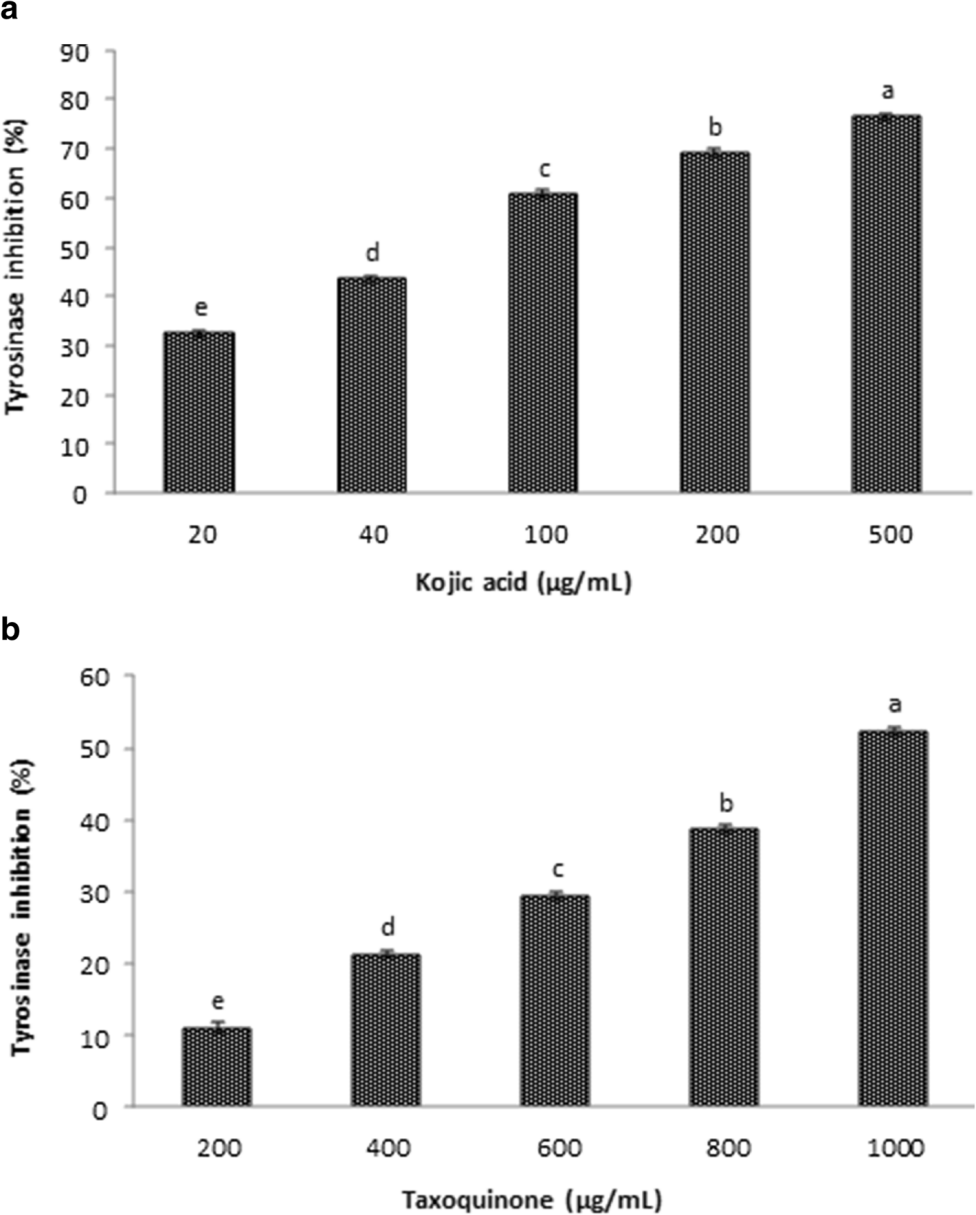
**Tyrosinase inhibitory effect of standard compound kojic acid (a) and taxoquinone (b) isolated from** ***Metasequoia glyptostroboides***. Data are expressed as mean ± SD (n = 3). Values with different superscripts are significantly different (p < 0.05).

The inhibitory activity of taxoquinone on the tyrosinase using a mushroom tyrosinase is demonstrated in Figure 3. In this assay, the taxoquinone at 200, 400, 600, 800 and 1,000 μg/mL showed 11.14, 21.33, 29.45, 38.65 and 52.32% of tyrosinase inhibitory effect, respectively. However, the mushroom tyrosinase inhibitory activity of standard compound kojic acid at 20, 40, 100, 200 and 500 μg/mL was found to be 32.41, 43.43, 60.81, 69.05 and 76.54%, respectively. Batubara et al. [16] observed that the phytoconstituent taxifolin and some other flavanonol rhamnosides from *K. malaccensis* also suppressed tyrosinase activity in the range of 5.86-25.95%*.* Piao et al. [17] demonstrated tyrosinase inhibitory effect of some chromones derived from a tropical plant aloe in a dose-dependent manner. In addition, the terpenoids isolated from the leaves of *Chloranthus tianmushanensis* were also found to exhibit tyrosinase inhibitor effect in a dose-dependent manner [18]. Similar findings on dose-dependent tyrosinase inhibitory activity of taxoquinone were observed in this study. Since it has been shown that tyrosinase inhibitors can repress the conversion of tyrosine to DOPA, dopaquinone and subsequently melanin, various tyrosinase inhibitors have been isolated and studied as potential candidates to decrease melanin content [19].

## Conclusions

In this research, a diterpenoid compound taxoquinone isolated from *M. glyptostroboides* demonstrated a considerable amount of α-glucosidase and tyrosinase inhibitory effects *in vitro* which may have potential to reduce after meal blood glucose levels and to maintain healthy skin by serving as a skin depigmentation agent. Based on the finding of this study, it can be concluded that taxoquinone in terms of its potent hypoglycemic and skin-whiting efficacy makes it to be a molecule of choice for using in health-care and drug therapies in the treatment of infectious diseases. However, further studies are needed to demonstrate a precise mode of action of taxoquinone to confirm its *in vivo* anti-diabetic and depigmentation potential.
